# Smart City Pilot Projects Using LoRa and IEEE802.15.4 Technologies

**DOI:** 10.3390/s18041118

**Published:** 2018-04-06

**Authors:** Gianni Pasolini, Chiara Buratti, Luca Feltrin, Flavio Zabini, Cristina De Castro, Roberto Verdone, Oreste Andrisano

**Affiliations:** 1 DEI, University of Bologna, Viale Risorgimento 2, 40136 Bologna, Italy; c.buratti@unibo.it (C.B.); luca.feltrin@unibo.it (L.F.); flavio.zabini2@unibo.it (F.Z.); roberto.verdone@unibo.it (R.V.); oreste.andrisano@unibo.it (O.A.); 2 IEIIT, National Research Council, Viale Risorgimento 2, 40136 Bologna, Italy; cristina.decastro@ieiit.cnr.it

**Keywords:** smart city, Internet of Things, testbed, LoRa, IEEE 802.15.4, measurements

## Abstract

Information and Communication Technologies (ICTs), through wireless communications and the Internet of Things (IoT) paradigm, are the enabling keys for transforming traditional cities into smart cities, since they provide the core infrastructure behind public utilities and services. However, to be effective, IoT-based services could require different technologies and network topologies, even when addressing the same urban scenario. In this paper, we highlight this aspect and present two smart city testbeds developed in Italy. The first one concerns a smart infrastructure for public lighting and relies on a heterogeneous network using the IEEE 802.15.4 short-range communication technology, whereas the second one addresses smart-building applications and is based on the LoRa low-rate, long-range communication technology. The smart lighting scenario is discussed providing the technical details and the economic benefits of a large-scale (around 3000 light poles) flexible and modular implementation of a public lighting infrastructure, while the smart-building testbed is investigated, through measurement campaigns and simulations, assessing the coverage and the performance of the LoRa technology in a real urban scenario. Results show that a proper parameter setting is needed to cover large urban areas while maintaining the airtime sufficiently low to keep packet losses at satisfactory levels.

## 1. Introduction

Infrastructure aging, vehicular mobility, energy saving and personal safety are some of the issues that place serious strains on the economic sustainability of human activities, as well as the quality of life in modern urban areas. As a matter of fact, the livability of medium and big cities has been deteriorating significantly in the last decades, and it is expected to get worse in the future. Indeed, projections indicate there will be more than 41 mega-cities (with more than 10M people) by 2030, with a relevant increase with respect to the current 28 mega-cities. At the same time, the percentage of population residing in urban areas is expected to reach 66% by 2050, compared to a figure of 54% in 2014 [1].

In recent years, the smart city paradigm has emerged as a revolutionary approach to tackle the challenges posed by the increasing complexity of urban environments [2]. By leveraging on Information and Communication Technologies (ICTs), smart cities promise to bring wide-ranging improvements to urban living, making the use of physical infrastructures (residential and public buildings, roads, utilities) more efficient, properly adapting to evolving circumstances, collecting valuable information for decision making processes and engaging effectively citizens in local governance. The expected reward of such a process is an inclusive environment where, thanks to innovative technical solutions, citizens experience healthy, safer and more efficient conditions.

Turning this vision into reality requires the integration of urban infrastructures, citizens and governance in a comprehensive and holistic perspective, which envisions the different components of the urban system as a unitary living body. In this biologic metaphor, ICTs play the role of the nervous system, connecting the different parts (infrastructures, people, governance) and providing the computational capability (i.e., the brain) for data analysis, features extraction and decision-making support.

The Internet of Things (IoT) paradigm represents the core of this scenario, as plenty of connected devices will operate in order to sense the physical world as well as to adapt the context to changing circumstances. In most cases, connectivity will be ensured by wireless networks, as they enable the exchange of information with a flexible and low-cost deployment. In this regard, a heterogeneous communication technology and different network architectures can be adopted, depending on the characteristics of the service to be implemented (e.g., low bit rate or high bit rate), operational constraints (e.g., availability of an external power source), coverage (e.g., long-range or low-range links), etc.

Mesh networks and short-range communication technologies, suitably integrated with cellular networks, are preferable, for instance, where end-to-end communications are not possible owing to power limitations and/or propagation obstacles. In this case, hop-by-hop communications from device to device can be exploited to convey information from the control center to the edge of the network, and backward. In this respect, different technologies can be adopted, such as IEEE 802.11s [3] and IEEE 802.15.4 [4]. The former is an amendment to the classic IEEE 802.11 standard (the so-called WiFi), introducing mesh networking and targeting bandwidth demanding services in scenarios with no energy consumption constraints. In contrast, IEEE 802.15.4 (which is the basis for ZigBee [5], ISA100.11a [6] and WirelessHART [7]) targets low bit rate communications among nearby devices with low energy consumption.

Star networks and long-range communication technologies are preferred, instead, when direct and reliable links between the central hub and all nodes deployed in its coverage region are available. This topology is adopted by most Low-Power Wide-Area Networks (LPWANs), which are especially designed to interconnect battery-powered devices with low bit rates over long ranges. Due to their low cost, wide coverage and straightforward setup, LPWANs are actively considered by academia and industry as the future wireless communication standard for IoT. Indeed, according to [8], by 2025 almost half of wide area IoT connections will be supported by LPWANs.

LPWANs operate on both unlicensed and licensed frequency bands and include proprietary and open standard options, among which the most relevant are LTE Cat-M1 [9] (also known as LTE-M), LTE Cat-NB1 [10] (also known as NB-IoT), FlexNet [11], LoRa [12], Sigfox [13] and Telensa [14]. In particular, LoRa will be the first LPWAN technology to break the milestone of 100 million connections, by the end of 2018 [8].

Both the above discussed solutions (short-range and long-range communications technologies with mesh and star topologies, respectively) are addressed in this paper, which presents the implementation of two smart city testbeds developed in Italy. The first one was developed in 2012 by the Wireless Communication Laboratory at the University of Bologna -WiLAB-, and operated until 2014 in the framework of the PEGASUS project. It consisted of the realization of a mesh topology (implemented on top of the IEEE 802.15.4 standard) of *enhanced* street lamps, which enabled smart services such as smart lighting, smart parking, vehicle-to-roadside communications and telemetering. The choice of the mesh network topology, along with the IEEE 802.15.4 short-range technology, is motivated by the regular deployment of light poles, usually at short distance one to the other. A successive real-world, large-scale implementation, which counts around 3000 street lamps installed in the Italian municipality of Montechiarugolo, is also presented in this paper. To the best of the Authors’ knowledge, this is the first paper showing a large-scale wireless network of street lights, which is fully operational since 2014.

The second testbed concerns with the implementation of a LPWAN in the framework of the RIGERS project, carried out with the support of the municipal council of Bologna, which is aimed at building-monitoring applications. In this case, the adoption of the IEEE 802.15.4 would be highly critical because of its reduced coverage, coupled with the difficult propagation conditions of indoor environments (nodes are placed inside apartments, which has a detrimental impact on the connectivity owing to the limited link budget of the IEEE 802.15.4 technology). For this reason, the long-range LoRa technology, which exhibits a high receiving sensitivity, has been chosen, along with the star network topology.

The project is still ongoing and this paper reports about: (1) the results of the measurement campaigns carried out in two different districts of the city of Bologna to estimate the related path-loss models; (2) the network-level performance obtained through a simulator incorporating the estimated path-loss models.

Apart from providing the technical details of the two testbeds and discussing their performance through experimental results and simulations, in this paper we want to highlight that, although addressed at the same urban scenario, IoT-based services as those considered here could require different technologies and network topologies in order to be effective. Indeed, owing to their differences (number of nodes, nodes layout, propagation conditions, etc.) the two testbeds are somehow orthogonal.

The paper is organized as follows. In Section 2 an overview of related work is provided. In Section 3 the communication technologies adopted in the testbeds are introduced. In Section 4 the smart lighting testbed is described, whereas the RIGERS project is discussed in Section 5. Final conclusions are drawn in Section 6.

## 2. Related Work

Smart City-based electronic products and applications are gaining importance nowadays. They address, for instance, urban mobility, public transportation, e-governance, safety, security, public lighting and environmental monitoring. Such complex scenarios are currently investigated by the Research Community. With reference to IoT deployments in smart city scenarios, a survey on research opportunities and challenges is provided in [15], and authors [16] discusses enabling technologies, weaknesses and risks. A survey on technologies and smart city applications is also reported in [17].

One of the objectives of the smart city concept is to monitor, control and manage the resources, such as the electric power. At this regards, the starting point for many current smart city implementations in Europe is the public lighting system [18,19]. Authors [20] focuses on the design of street light controllers to provide a reduction in power consumption and eventually a reduction in cost of public lighting. The paper provides an estimation of the energy saved but does not report about real values of the power consumption drop achieved through a real deployment, as done here. Moreover, in contrast to this paper, current literature dealing with smart public lighting either shows simulation results (e.g., [21,22]) or presents the results of limited testbeds (few nodes) (e.g., [21,23,24]).

As far as the adoption of the IEEE 802.15.4 technology in smart city scenarios is concerned, several papers are currently available. As an example, authors [25] discusses a simulator for evaluating the performance of IEEE 802.15.4 and IEEE 802.11 when applied to such scenarios. Authors [26] investigates the deployment of wireless sensor networks at road intersections in some of the world major cities. Using a propagation model that corresponds to the 2.4 GHz IEEE 802.15.4 technology and considering 52 city maps extracted from OpenStreetMap, the Authors show that the resulting graph is highly disconnected and comprises up to 25% of isolated nodes. However, in the above cited works and in most of the papers on this topic, either no experimental results are provided or very limited testbeds are investigated. As far as significant testbeds are concerned, one of the largest is Smart Santander [27], where around 3,000 IEEE 802.15.4 devices were deployed, which makes the size of such testbed similar to the one presented in this work. However, in contrast with this paper, authors [27] does not deal with smart lighting, thus no specific insight is provided on such application nor on the corresponding economic benefits.

With regard to the adoption of LoRa in smart city scenarios, authors [28] presents a LoRaWAN demonstrator to be deployed at the Scientific Campus of the University of Lille. Prior to the installation of the LoRaWAN network, a field study was carried out to explore the link-level performance of such technology. Tests were conducted in both outdoor and indoor environments using only one gateway and showed that LoRa provides good performance over the major part of the Campus. However, neither capacity nor packet success rate were evaluated in the paper. In [29] the authors compare via experimentation the link-level performance obtained with the LoRa proprietary modulation and with an FSK (Frequency Shift Keying) modulation. Experiments are performed in an urban environment and results suggest the superior performance of LoRa over FSK under adverse transmission conditions. Authors [30] studies the performance of a LoRa-based IoT network in a typical urban scenario. The Authors provide simulation results showing that a LoRa network can scale well, thanks to the possibility to use multiple gateways and be equipped with different radios, capable of receiving signals transmitted with different spreading factors at the same time. Finally, authors [31] shows results obtained through the NS-3 network simulator, where the bit error rate, together with the LoRaWAN MAC protocol, are modelled. Results concerning single and multiple gateways scenarios are reported, such as the impact of the spreading factor on the performance. However, the paper does not deal with smart city scenarios and does not incorporate realistic channel models based on measurements, as done in this paper.

Authors [32] presents results achieved through a small testbed composed of two LoRa devices equipped with different sensors (light, temperature, smoke, humidity, etc.), which sends measurements to a gateway placed in the university campus. The Authors do not characterize the network performance neither from a link- nor from a network-level viewpoint, but they just show data measured by sensors to demonstrate the feasibility of the testbed. Similar considerations can be done for [33], which reports a PoC deployment addressing the problem of river Liffey monitoring in Dublin city center. They deployed a buoy on the Liffey river in Dublin for a duration of 8 months. Also, in this case, the results consist of measured data in terms of water temperature, atmospheric pressure and water depth.

In contrast with the above cited works, in this paper we consider a real, densely built-up, urban scenario, where devices are located inside buildings. First, we derive the path-loss models for two districts of Bologna through in-field measurements, then we use such models in a network simulator allowing to derive the LoRa performance at network-level.

## 3. Technologies

### 3.1. IEEE 802.15.4 Technology

IEEE 802.15.4 [4] is a short-range wireless technology supporting applications with relaxed throughput and latency requirements in wireless personal area networks (PANs). The key features of 802.15.4 are low complexity, low cost, low power consumption, and low data rate transmissions. Each PAN works on a different channel and is managed by a coordinator, gathering data from devices in the PAN.

As far as the physical layer is concerned, in this paper we consider the 2.4 GHz band physical layer, characterized by a bit rate of 250 kbit/s and by a minimum shift keying modulation on top of which direct sequence spread spectrum is applied.

As for the medium access control (MAC) protocol, we consider the non beacon-enabled mode, where nodes may access to the channel in a distributed way (without the need of being synchronized to the coordinator), using an unslotted carrier sense multiple access with collision avoidance (CSMA/CA) protocol [4].

One important operational aspect of the IEEE 802.15.4 technology is its ability to define different types of devices: Reduced Function Devices, which have limited capabilities and cannot forward data, and Full Function Devices, that can act as dynamic routers in a mesh topology. Different solutions may be adopted at the network layer to create mesh topologies (e.g., Zigbee, 6LoWPAN, etc.); in this work we used a proprietary routing algorithm inspired by the Many-to-One routing strategy defined in Zigbee.

This is the technology we adopted to implement the smart lighting testbed described in Section 4, which is based, in fact, on a mesh network among smart street lamps.

### 3.2. LoRa Technology

LoRa [34,35] uses a proprietary modulation based on chirp spread spectrum, which exploits chirps whose frequency increases or decreases linearly over a certain amount of time; information is inserted in chirps by introducing a frequency discontinuity at different time offsets [36]. These chirps occupy a bandwidth of 125, 250 or 500 kHz. One of the most important parameters of the physical layer is the Spreading Factor (SF), which is the ratio between the signal bandwidth and the symbol rate. Keeping the bandwidth constant, it is possible to improve the receiver sensitivity by increasing the airtime (duration of a packet transmission). More precisely, each increment by one unit of the SF, corresponds to a doubling of the airtime and a decrement of the receiver sensitivity of roughly 3 dB.

At MAC layer LoRa uses the LoRaWAN protocol that describes three Classes: (i) Class A: end devices, after the transmission of a packet, open two receive windows to get an acknowledgement (ACK) or receive data from the gateway, then they stay in idle mode until the next transmission; (ii) Class B: end devices have more receive windows synchronized with a beacon provided by the gateway; (iii) Class C: end devices stay continuously in reception mode. Any device has to be compliant with, at least, Class A (considered in this paper), where ALOHA protocol is used in uplink.

There are two types of transmission modes: confirmed, using ACKs, and unconfirmed, when no ACKs are used. When an ACK is expected but not received by the transmitter, a recovery algorithm, which consists in multiple retransmissions, is initiated. In case the gateway is able to receive simultaneous signals transmitted with different spreading factors, the standard suggests that every two failed retransmissions the data rate is decremented, resulting in an increase of SF and of the airtime, as well. Indeed, this algorithm implicitly assumes that the transmission failed due to poor connectivity, therefore a lower data rate should increment the success rate, as the receiver sensitivity will be better. However, if losses are due to collisions, such strategy does not mitigate transmission failures, as SF continues to be increased, resulting in longer airtimes and, ultimately, in a larger collision probability.

As for network aspects, LoRaWANs are generally laid out in a star topology and the central node is usually called *gateway*.

This is the technology adopted within the RIGERS project described in Section 5, which is actually based on a star network aimed at collecting data sensed by devices located inside and outside selected buildings in Bologna.

## 4. Smart Lighting Testbed

In the framework of the PEGASUS project, funded by the Italian Ministry for Economic Development, WiLAB realized an experimental testbed at the premises of Fondazione Alma Mater (University of Bologna), where a network of smart, remotely controlled street lamps was deployed, which was also capable of supporting the provision of smart services.

**Figure 1:**
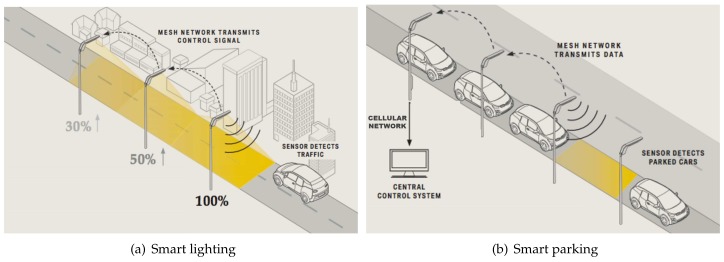
Examples of smart services enabled by intelligent street lamps.

The main objective was twofold. First, the poles wireless network was meant to increase the efficiency of the lighting infrastructure itself, reducing electricity consumption via a combination of dimmers and task lighting (Figure 1a). Second, it was meant as an access network for the provision of context services, such as smart parking (Figure 1b).

**Figure 2:**
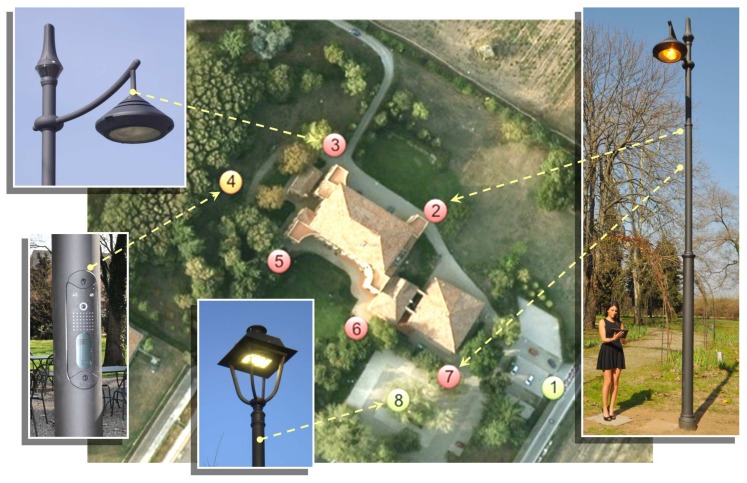
Smart city testbed at Fondazione Alma Mater (2012).

More precisely, eight LED street lamps located in the park of the historic building Villa Gandolfi Pallavicini (Bologna), shown in Figure 2, were equipped with IEEE 802.15.4 short-range communication interfaces, on top of which the proprietary routing protocol implemented at the University of Bologna and allowing to create a mesh topology, was implemented. One of the poles (hosting the coordinator) was also capable of long-range communications, through a cellular interface, which allowed the connection of the whole pole PAN with a remote control center. All the poles were thus completely managed by the control center with no need for a fixed communication infrastructure, allowing the remote dimming of each light fixture independently of the others, as well as the monitoring of their operating conditions through individual telemetering (e.g., power consumption and temperature) and telediagnostic.

The deep expertise gained from the deployment and management of such testbed led to a large-scale implementation of the smart lighting technology in the Italian municipality of Montechiarugolo. Here, the whole public lighting infrastructure, around 3000 luminaries in a 211 square kilometres area, was enhanced accommodating LED lamps and the devices needed for establishing wireless communications and the provision of smart services. This infrastructure is fully operational since 2014, when the replacement of the previous fixtures (mainly working with sodium-vapor lamps) was completed.

**Figure 3:**
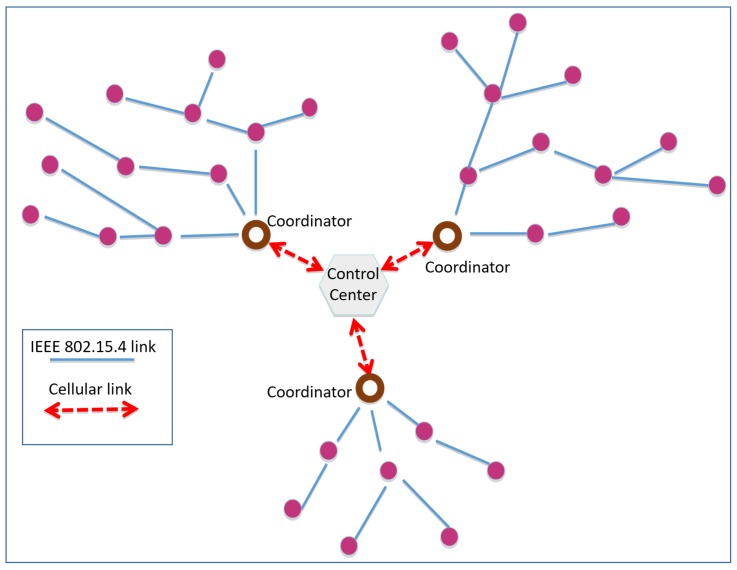
Montechiarugolo: exemplification of the network architecture.

All poles of the new infrastructure are equipped with the circuitry for the dynamic light dimming and with IEEE 802.15.4 communication interfaces (for the mesh network establishment), having a transmission power of 20 dBm and a transmission rate of 250 kbit/s. Exchanged packets have a maximum payload length of 64 bytes. From a hierarchical point of view, the network is organized in clusters (i.e., PANs) of 100∼150 light poles (depending on propagation conditions), each one managed by a coordinator. The number of hops between the coordinator and the cluster peripheral goes from 5 to 10 (depending on the cluster topology), which determines in the worst case a latency from the control center to the network border of 100 ms, more than adequate for smart lighting applications. Moreover, in order to minimize inter-cluster interference, different channels were chosen for adjacent clusters. Figure 3 shows an exemplification (with only three clusters and 30 nodes) of the network architecture.

In perspective, all poles are also ready to host additional sensors, in order to allow, for instance, environmental monitoring or motion detection for context-aware light dimming.

Thanks to the wireless network, the light intensity of each street lamp can be remotely controlled with no need of a fixed communication infrastructure. Moreover, the switching and dimming of street lights can be remotely programmed and re-programmed as per requirement so as to save valuable power. Lamp diagnostic, real-time fault detection and energy consumption controllers are implemented as well, providing city managers with all the information required for an efficient service provision.

**Figure 4:**
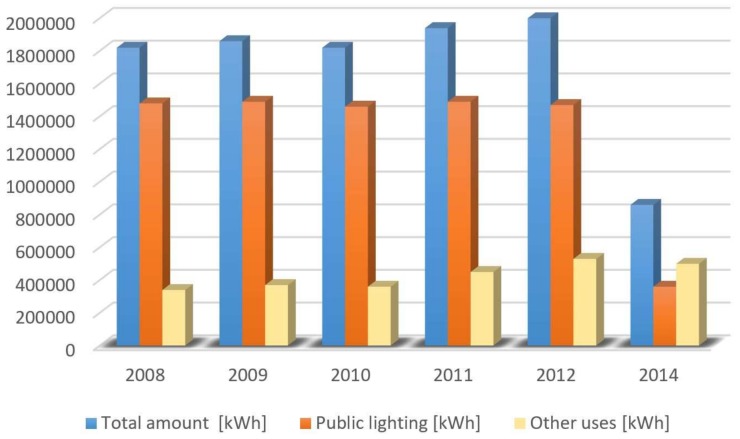
Impact of the smart lighting technology in Montechiarugolo.

Figure 4 shows the dramatic economic impact that the smart lighting technology had in Montechiarugolo. In 2014, the first year of life of the new public lighting infrastructure, the power consumption dropped from 1,500,000 kWh to 358,000 kWh (−76%), with a significant money saving (−73%). In addition, the joint effect of LED lamps and wireless remote control led to a saving of 224 TEP (tonne of oil equivalent) in a year and avoided the emission, in the same period, of 500 tons of ${\mathrm{CO}}_{2}$.

Most notably, the free access to such network could be granted also to private citizens, as it happens in Montechiarugolo. Here, the owners of business activities with high lighting costs (e.g., firms, sports centers and farms) are encouraged to adopt smart lighting technologies by allowing them to freely connect to the public mesh network as well as by freely providing them with the software for the management of the private lighting infrastructure. In exchange, the municipality receives from private citizens the white certificates (a white certificate is an instrument issued by an authorized body guaranteeing that a specified amount of energy savings has been achieved. Under such a system, producers, suppliers or distributors of electricity are required to undertake energy efficiency measures that are consistent with a pre-defined percentage of their annual energy deliverance. If energy producers do not meet the mandated target they are required to pay a penalty. As white certificates are tradable, those who have overcomplied can sell their certificates to those who have not met their obligations.) which are granted by the Italian Energy Authority. Such certificates are then sold by the municipality, along with those that Montechiarugolo gains by itself thanks to its smart lighting infrastructure. Therefore, this mechanism provides an additional income for the municipality.

However, the benefits of such technology are not limited to economic aspects. A relevant consequent effect is the availability of a capillary wireless network, which reaches every location where a smart street lamp is present. This enables the collection of data and the provision of new services, concerning for instance

- *smart parking*: real time monitoring of parking lots (vacant/occupied);
- *waste management*: detection of rubbish levels in containers to optimize the trash collection routes and schedule;
- *air quality*: monitoring of the air pollution;
- *structural health*: monitoring of vibrations and material conditions in buildings, bridges and historical monuments;
- *vehicular traffic*: detection of traffic jams and suggestion of alternative routes through variable message signs.

In particular, the smart parking service has been tested in Montechiarugolo, where a few parking lots have been equipped with magnetometers buried under the street surface. The magnetometer was capable of revealing the magnetic field change caused by the presence of a vehicle and transmitting the parking lot status to the nearest light pole. Short range communications between the magnetic sensors and the light pole took place by means of the Micro.Sp™ technology, patented by STE Industries, which has been expressly designed for applications where the battery duration (the sensing device and the communication equipment are battery powered, hence the power consumption is a critical issue. Micro.sp™ devices are supplied by 3V lithium battery for a duration of over 10 years.) is a critical aspect. Micro.Sp devices operated in the 169 MHz band with the pulse position modulation (PPM) and periodically transmitted the parking lot status, the magnetometer ID and the battery level. Such data were then forwarded by the “receiving light pole”, equipped with the Micro.sp technology, to the cluster coordinator through the 2.4 GHz mesh network. Finally, the coordinator forwarded these data to the control center by means of the cellular network (Figure 1b).

The whole system has been successfully tested, however no smart parking service has been activated yet, because it is not among the highest priorities of the municipality. Indeed, activating such service would require further significant investments for the capillary deployment of magnetic sensors and, to a lesser extent, for the implementation of the hardware/software platform in charge of conveying to drivers, through the cellular network, the parking lot availabilities.

## 5. A Smart City Testbed: The Rigers Project

The RIGERS project is aimed at monitoring residential and public buildings in two districts of Bologna, called Saragozza and Navile, for a time period of one year. Different rooms of each monitored building will be equipped with a multi-sensor platform using a LoRa transceiver to communicate the measured data to the control center. In particular, humidity, temperature, lighting, CO_2_ sensors and a globe thermometer will be installed in each platform. Each of these sensors will periodically (every five minutes) take a measurement from the environment and every hour the average of the measured values will be sent to the LoRa gateway via single hop (star topology). The gateway will then forward the received data to the control center via a 3G network.

The RIGERS project is still ongoing and the final deployment has not been carried out yet. As a result, in this section we report: (1) the experimental derivation of the path-loss model in the two districts of Bologna cited above; (2) the assessment of the network-level performance through a simulator implementing the real final scenarios and incorporating the derived path-loss models.

### 5.1. Path-Loss Characterization via Experimentation

A preliminary testbed was realized at WiLAB to get an insight on the actual performance of a LoRa-based LPWAN deployed in the Saragozza district of Bologna. In particular, two Embit Development boards EMB-LR1272-EVK hosting the LoRa module EMB-LR1272 were adopted. Each module was equipped with a half-wavelength dipole antenna with a gain of 2 dB.

**Figure 5:**
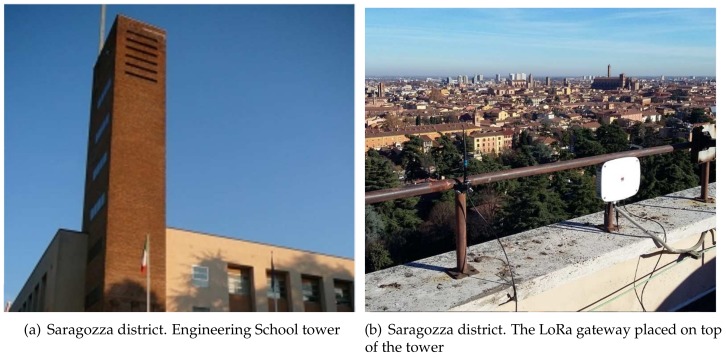
LoRa gateway setup.

One of them, acting as gateway, was placed on top of the Engineering School tower at a height of 71 m above the ground, as shown in Figure 5, whereas the second device, powered by batteries and equipped with a GPS receiver, was carried around in the city at a pedestrian speed, 1.5 m above the ground.

Every 8 s, the mobile terminal transmitted to the gateway one packet, whose payload carried the device coordinates, with an output power of 14 dBm (the maximum allowed by the board) in a 125 kHz bandwidth centered on 868.1 MHz. The spreading factor was set to 12 (the highest possible choice), to improve the receiver sensitivity.

Upon the reception of each packet, the gateway i): recorded the correspondent Received Signal Strength (RSS), along with the coordinates of the transmitting device, and ii) responded with an ACK packet, to inform its counterpart that it was within the coverage range.

A total amount of 2425 RSS measurements was thus collected, with a maximum distance from the gateway of 2390 m. For larger distances, in fact, the received power was below the devices sensitivity, making communications almost or completely impossible.

**Figure 6:**
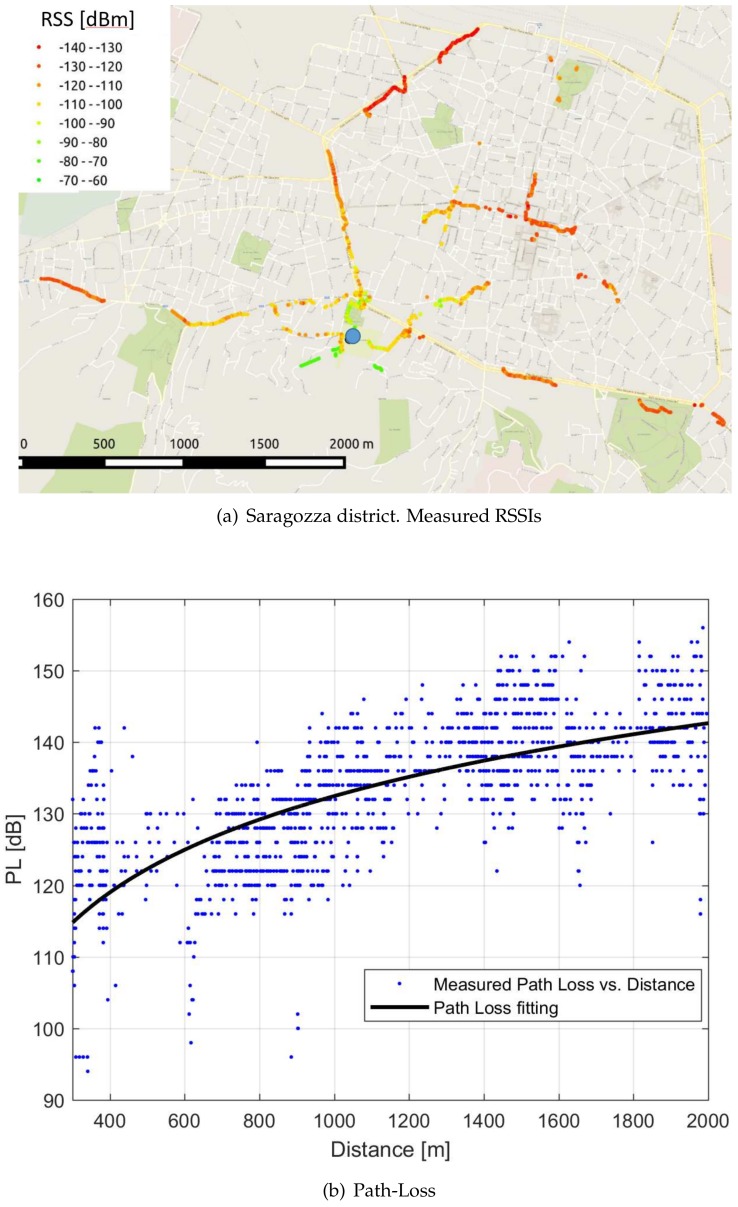
Saragozza district. Outcomes of the measurement campaign.

The outcome of the measurement campaign is summarized in Figure 6a, which provides a pictorial representation of the RSS values experienced by the gateway. In particular, the blue circle denotes the gateway location, whereas the colored dots (colour shades from green to red) represent the positions of the transmitter along the traveled paths. The specific color of a dot in a given position is representative of the RSS experienced by the gateway when a device was transmitting in such position. It is worth observing that, despite the favorable position of the gateway (71 m above the ground level), which resulted in propagation conditions cleared from obstacles for most of the signal path, the service coverage is around 2.5 km, which is below the distance that LoRa devices are supposed to cover. This is due to the low elevation of the mobile device (1.5 m from the ground), which suffered from the shadowing introduced by nearby buildings.

This phenomenon is well evident in Figure 6b, where the blue dots represent the path-loss measured in correspondence of the transmitter-receiver distance reported in the abscissa. The solid curve refers to the path-loss law PL that best fits the measurements, assuming for PL [dB] the one-slope model [37]

(1) $$PL\left[dB\right]=PL_{0}\left[dB\right]+10\alpha \log\left(d\right),$$

where

- *d* is the distance between the transmitter and the receiver in meters,
- $PL_{0}$ is the path-loss at 1 meter distance, whose measured value is 31 dB,
- α is the power decay index.

In the specific case plotted in Figure 6b, the best-fit curve has been obtained with α=3.25. This value, quite far from the ideal (free-space) value of 2, confirms the relevant impact of obstacles nearby the mobile device, despite the fact that the signal experiences free space propagation for most of the flying time.

Even more relevant is the path-loss spread that can be observed in Figure 6b for a given distance (vertical spread). This phenomenon is summarized by the Root Mean Square Error (RMSE) with respect to the best-fit curve, which is 7.28. This value, quite large indeed, confirms once more the significant role played by the surroundings of the mobile terminal for the experienced performance.

In view of the RIGERS project, it is, therefore, of paramount importance a proper choice of the gateway position, especially considering that most of LoRa devices will operate indoor, with additional propagation losses.

The above experiments have been replicated in the Navile district as follows. The gateway was located in the municipality building at 44 meters from the ground, while the LoRa device was located in different outdoor positions at 1 meter from the ground. Assuming the same one-slope path-loss model (1) adopted in the Saragozza district, the best-fit has been obtained with α=3.84.

The experimental path-loss models derived above will be adopted in Section 5.2 to assess the success rate of point-to-multipoint LoRa transmissions in the two districts.

### 5.2. Network-Level Performance Assessment via Simulations

In view of the in-field implementation of the LoRa network envisioned within RIGERS, in this section we present network-level results obtained through a proprietary simulator incorporating the above derived path-loss models. The simulator, written in C++, has been developed by the University of Bologna in collaboration with TIM (TIM -Telecom Italia Mobile- is, along with Vodafone, the main Italian telecommunications company, which provides telephony services, mobile services, and DSL data services.). Obtained results have been qualitatively compared with those presented in [31], where the NS-3 simulator, modelling the LoRa bit error rate and the LoRaWAN MAC layer, has been used. In contrast with [31], in this work the behavior of a single link in terms of device sensitivity and packet capture effect have been characterized experimentally. However, a complete experimental validation of the network-level performance is difficult to carry out, due to the number of devices that should be deployed in order to observe a reasonable number of collisions.

Simulation parameters are provided in Table 1, while receiver sensitivities and bit rates for the case of 125 kHz bandwidth, when setting different spreading factors, are reported in Table 2. The results reported in this section have been obtained by averaging over 1000 different scenarios (i.e., nodes distributions in the area, see below), and transmitting 100 packets per device in each scenario.

**Figure 7:**
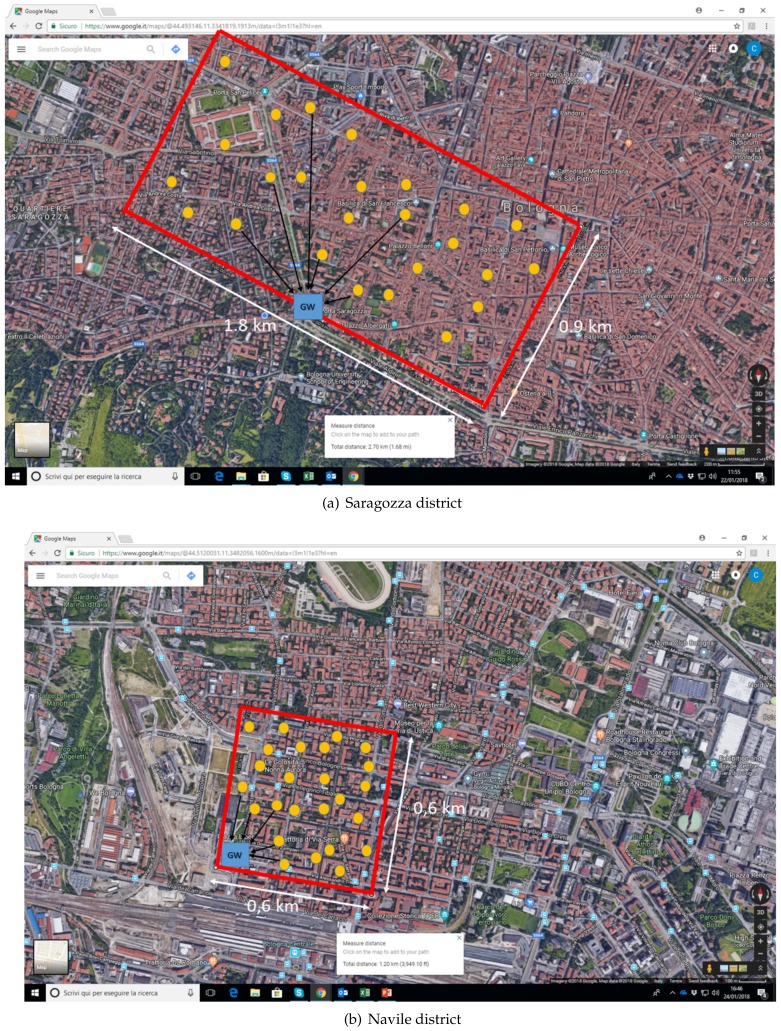
Simulated reference scenarios.

The simulated scenarios are the Saragozza and Navile districts of Bologna. In the former case we investigated a rectangular area of 0.9 km × 1.8 km, with the gateway located as reported in Figure 7a, while in the case of the Navile district, shown in Figure 7b, a square area of side 0.6 km was considered, with the gateway located in the corner of the area.

In each simulated scenario, *N* buildings were randomly chosen with uniform distribution in the area, and each building was assumed to be equipped with one multi-sensor board (Figure 7 shows an example of simulated snapshot and devices location when 25 devices are deployed).

According to the RIGERS application, every five minutes the board is switched on and all the sensors take a sample. Measured values are then averaged every hour and coded into a single packet transmitted to the gateway through the LoRaWAN access mechanism. Since each measurement take 2 bytes, each multi-sensor board generates 10 bytes (2 bytes by five sensors) of payload every hour. In the simulator, a packet is detected by a receiver (the gateway in our case), if the received power, $P_{r}$ given by Equation (2), is greater than the receiver sensitivity (whose values are reported in Table 2 for the different SF). If this condition is fulfilled, the receiver locks to the preamble and tries to decode the payload. When a packet collision occurs, the signal-to-interference ratio (SIR) is calculated (as the ratio between the power received over the useful link and the sum of the interfering powers) and the payload is passed to the upper layers provided that the device is locked to that particular packet and that the SIR is greater than the protection ratio, γ. An acknowledgment mechanism is also used and up to three retransmissions per packet are allowed.

In order to compute the received power, $P_{r}$ [dBm], the following model is used:

(2) $$P_{r}=P_{t}+G_{t}+G_{r}-PL-PL_{\mathrm{add}}+s,$$

where $P_{t}$ [dBm] is the transmit power, $G_{t}$ [dB] and $G_{r}$ [dB] are the transmit and receive antenna gains and PL [dB] is given by Equation (1). $PL_{\mathrm{add}}$ is the penetration loss: since the gateway will be located on the roof of a building and the multi-sensor boards will be deployed indoor, this factor accounts for the additional loss, due to the penetration of the wall. Finally, *s* is a Gaussian random variable having zero mean and standard deviation σ, representing shadowing.

**Figure 8:**
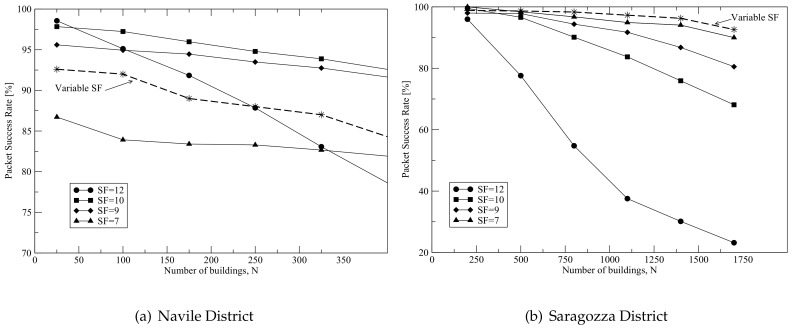
Packet success rates vs. number of buildings in both the Navile and Saragozza districts.

We consider two cases: (i) gateway equipped with a single receiver, which can be tuned to a specific channel (125 kHz bandwidth) and synchronized on a specific spreading factor value; (ii) gateway equipped with different receivers, such that it is able to decode signals transmitted using different spreading factors (again a single channel of 125 kHz bandwidth is considered). The first case refers to the receiver that will be used in the RIGERS project, while the second case refers to a LoRaWAN gateway. In the second case, as stated in Section 3.2, all nodes start using SF=7 and then, every two retransmissions, SF is increased by one to mitigate coverage issues (curves related to this case are denoted as “variable SF” in Figure 8). After three hours of simulation, SF is reset to 7.

Figure 8a,b report the percentage of packets correctly received by the gateway (packet success rate—PSR), as a function of the number of buildings deployed, *N*, for the Navile and Saragozza districts, respectively. Both the cases of variable and fixed SF, when considering different values of SF, are reported in the figures.

In the case of Navile, when setting low values of SF, the network is limited by connectivity and the PSR slightly depends on *N*. Indeed, the ideal (when shadowing is not present) transmission range is within 0.3 km (obtained when setting SF=7) and 0.8 km (for the case SF=12), while the maximum distance covered in the scenario is 0.85 km. This connectivity problem is mitigated when setting SF = 12, allowing to reach more buildings (lower receiver sensitivity); however, as SF increases, the airtime increases as well (1.18 s when SF = 12, versus 0.05 s when SF = 7), resulting in a larger collision probability and a reduction of the PSR when *N* increases. Since nodes use a simple ALOHA protocol to access the channel, an increase in airtime brings to a performance worsening in interference limited scenarios. In conclusion, SF = 10 is the best case, allowing a good trade-off between connectivity and collision issues, and allowing to achieve a PSR larger then 90% for up to 400 nodes/buildings. In this scenario, the strategy suggested by the standard to dynamically set SF (variable SF) does not allow to obtain the best performance. This is due to the fact that the scenario is limited by connectivity, therefore many nodes do not receive the ACK, increase SF and after some transmissions many nodes tend to use SF=12, which is not the best value because of collisions (see the curve SF=12). Finally, note that the trends of the curves reported in this figure are very similar to those of the curves reported in Figure 9 of [31]: in particular, a weak dependence of the packet delivery ratio on the number of devices is observed also in [31] in case of connectivity issues (almost flat behavior of the curve related to SF=7). Moreover, also in [31] the choice SF=12 appears the best solution when few nodes are present, but it becomes the worst case when increasing the traffic generated, because of collisions.

As far as Saragozza is concerned, being the power decay index lower, there are not connectivity issues: a PSR of 100% is obtained for low values of *N*. As a result, the PSR decreases with *N* for all values of SF and SF=7 is the value that maximizes the success rate, since it is characterized by the lowest value of airtime. In this case, if the application requires a minimum PSR of 90%, the LoRa gateway is able to serve up to approximately 1700 buildings. In this scenario, instead, the use of a variable SF allows to maximize the performance, because less connectivity issues are present and only few nodes increase SF. As a result, performance is better than in the case SF=7, since more nodes can reach the gateway, but just few nodes increase SF, therefore collisions are still mitigated.

In the above figures, the standard deviation of the PSR is always below 2%.

## 6. Conclusions

In this paper, two important applications envisioned in smart city scenarios have been addressed: public smart lighting and building-monitoring. The former has been discussed showing that, thanks to the regular layout of street light poles, usually at short distance one to the other, a mesh topology using IEEE 802.15.4 at PHY and MAC layers could be adopted, jointly with the cellular network, to grant the connectivity to all of them, even in the case of thousands of nodes deployed in a wide area. Not only does the paper show the feasibility of such a solution (providing the technical details) but it also gives an insight into the relevant benefits in terms of power and money savings (−76% and −73%, respectively, in the real world-case represented by the Italian municipality of Montechhiarugolo).

In the case of the building-monitoring application, instead, a star network topology has been considered, which is more suited to a scenario in which the nodes layout could be so irregular that in many cases nodes “do not see” each others, thus preventing the possibility of clustering.

In this case, the long-range LoRa technology has been taken into account and it has been experimentally shown that its maximum coverage in a dense urban environment is in the order of 1–2 km, which is well below the 15 km claimed by LoRa manufacturers and vendors. Remarkably, this result has been obtained in very favorable conditions, with the gateway placed at a height of 71 m above the ground and the highest possible spreading factor. Therefore, it is expected that the coverage could be even lower when such conditions cannot be granted. Moreover, this paper showed how to tune the spreading factor adopted by the transmitter, in order to maximize the packet success rate in multipoint-to-point communications. Results demonstrate that, in order to cover an entire district, it will be requested to deploy multiple LoRa gateways, or to equip the gateway with multiple receivers, working on different channels and using different spreading factors.

Future work: The two testbeds discussed in this paper are based on distinct wireless networks, expressly deployed for the specific services. In the next future, however, 4G cellular networks will also support the NB-IoT technology, which will be assigned a given amount of LTE resource blocks. In Italy, for instance, the LTE networks of the main operators will be enhanced with NB-IoT by the end of 2018. There is no guarantee that NB-IoT will outperform currently available short-range and long-range technologies; its introduction, however, will certainly change the IoT scenario in urban environments, as 4G networks are widespread, reliable and supported by big, in some cases supranational, operators.

Motivated by these considerations, we believe that future research activities in the field of urban-IoT for smart cities should pay proper attention to NB-IoT, which is candidate to become the de facto standard for machine-to-machine communications.

## Figures and Tables

**Table 1:** Simulation Parameters.

Parameter	Value
Transmit Power, $P_{t}$	14 dBm
Antenna gains, $G_{t}=G_{r}$	2 dB
Penetration Loss, $PL_{\mathrm{add}}$	12.5 dB
Shadowing standard deviation, σ	9
Protection ratio, γ	0.3 dB
Payload size	10 Bytes
Bandwidth	125 kHz
Frequency of packets generation	1 h

**Table 2:** Bit Rates and Receiver Sensitivities for the 125 kHz bandwidth.

Spreading Factor	Bit Rate (Bit/s)	Indicative Receiver Sensitivity [dBm]
12	250	−137
11	440	−135
10	980	−133
9	1760	−130
8	3125	−127
7	5470	−124
